# Nasal high flow reduces dead space

**DOI:** 10.1152/japplphysiol.00584.2016

**Published:** 2016-11-17

**Authors:** Winfried Möller, Sheng Feng, Ulrike Domanski, Karl-Josef Franke, Gülnaz Celik, Peter Bartenstein, Sven Becker, Gabriele Meyer, Otmar Schmid, Oliver Eickelberg, Stanislav Tatkov, Georg Nilius

**Affiliations:** ^1^Comprehensive Pneumology Center, German Center for Lung Research, Munich, Germany;; ^2^Institute of Lung Biology and Disease, Helmholtz Zentrum München–German Research Center for Environmental Health, Neuherberg, Germany;; ^3^Fisher & Paykel Healthcare, Auckland, New Zealand;; ^4^HELIOS Klinik Hagen-Ambrock, Witten-Herdecke University, Hagen, Germany;; ^5^Department of Nuclear Medicine, LMU Medical Center Grosshadern, Munich, Germany;; ^6^Department of Otolaryngology, Head and Neck Surgery, LMU Medical Center Grosshadern, Munich, Germany;; ^7^Department of Nuclear Medicine, Asklepios Fachkliniken München-Gauting, Gauting, Germany; and; ^8^University Hospital of the Ludwig Maximilian University, Munich, Germany

**Keywords:** nasal high flow, upper airways, dead space, rebreathing, Krypton, respiratory support

## Abstract

Recent studies show that nasal high flow (NHF) therapy can support ventilation in patients with acute or chronic respiratory disorders. Clearance of dead space has been suggested as being the key mechanism of respiratory support with NHF therapy. The hypothesis of this study was that NHF in a dose-dependent manner can clear dead space of the upper airways from expired air and decrease rebreathing. The randomized crossover study involved 10 volunteers using scintigraphy with ^81m^Krypton (^81m^Kr) gas during a breath-holding maneuver with closed mouth and in 3 nasally breathing tracheotomized patients by volumetric capnography and oximetry through sampling CO_2_ and O_2_ in the trachea and measuring the inspired volume with inductance plethysmography following NHF rates of 15, 30, and 45 l/min. The scintigraphy revealed a decrease in ^81m^Kr gas clearance half-time with an increase of NHF in the nasal cavities [Pearson’s correlation coefficient cc = −0.55, *P* < 0.01], the pharynx (cc = −0.41, *P* < 0.01), and the trachea (cc = −0.51, *P* < 0.01). Clearance rates in nasal cavities derived from time constants and MRI-measured volumes were 40.6 ± 12.3 (SD), 52.5 ± 17.7, and 72.9 ± 21.3 ml/s during NHF (15, 30, and 45 l/min, respectively). Measurement of inspired gases in the trachea showed an NHF-dependent decrease of inspired CO_2_ that correlated with an increase of inspired O_2_ (cc = −0.77, *P* < 0.05). NHF clears the upper airways of expired air, which reduces dead space by a decrease of rebreathing making ventilation more efficient. The dead space clearance is flow and time dependent, and it may extend below the soft palate.

**NEW & NOTEWORTHY** Clearance of expired air in upper airways by nasal high flow (NHF) can be extended below the soft palate and de facto causes a reduction of dead space. Using scintigraphy, the authors found a relationship between NHF, time, and clearance. Direct measurement of CO_2_ and O_2_ in the trachea confirmed a reduction of rebreathing, providing the actual data on inspired gases, and this can be used for the assessment of other forms of respiratory support.

recent studies report that an open nasal cannula system that generates nasal high flow (NHF) with or without supplemental oxygen (O_2_) can assist ventilation in patients with chronic respiratory failure (1, 5, 22, 24) or sleep disorders (17, 21), in hypoxemic patients after cardiothoracic surgery, and in those with acute hypoxemic respiratory failure (6, 25, 28). In addition, the use of this form of respiratory support in pediatrics and in newborns has proven clinical benefits (8, 11, 15). Delivering a high flow of gas through the open nasal cannula to generate airway pressure (27) has been tried in the past, but developments in technology have now allowed efficiently heated and humidified respiratory gases to enable a wide range of flow rates from 2 l/min in preterm newborns to 60 l/min in adults (24, 28).

A number of clinically relevant benefits have been associated with NHF therapy: reduction in respiratory rate, a decrease of minute ventilation during sleep, improved alveolar ventilation, and a reduction in wasted ventilation and the work of breathing (4, 11, 23, 28), although how NHF produces these effects is not yet understood. A mechanistic study on healthy volunteers suggested two different ventilatory responses to NHF, one when awake and another during sleep (19). In this study it was speculated that the reduction of dead space ventilation due to clearance of anatomical dead space in the upper airways could be the principal driver for the reduction of minute ventilation during sleep, which may potentially lead to a reduction in the work of breathing. In a previous study using upper airway models the authors demonstrated the fast-occurring flow-dependent clearance of nasal cavities by NHF (18). Dead space clearance is difficult to study in vivo because of the complexity in quantifying the respiratory gases in the airways. However, many have proposed it to be the major physiological mechanism that improves respiratory support (20, 22, 26) and reduces arterial and tissue CO_2_ (1, 7, 14).

The aim of this study was to measure upper airway dead space reduction during NHF therapy to test a hypothesis that NHF in a dose-dependent manner can clear dead space in the upper airways and decrease rebreathing.

Clearance of ^81m^Kr tracer gas from the upper airways by NHF was assessed in healthy volunteers using dynamic gamma camera imaging. Reduction of rebreathing was investigated in tracheotomized patients using volumetric capnography and oximetry by sampling gas from the trachea while the patients maintained nasal breathing during NHF therapy.

## METHODS

### Study participants.

Ten healthy, nonsmoking volunteers [age 55 ± 14 (standard deviation, SD) yr] participated in the tracer gas scintigraphy study (Table 1). This part of the study was approved by the Ethics Committee of the Medical School of the Ludwig Maximilian University (Munich, Germany), and written informed consent was obtained from each subject.

**Table 1.** Anthropometric data of 10 healthy volunteers participating in the study

	Value
Male/female	7/3
NS/S/XS	7/0/3
Age, yr	55 ± 14
Height, cm	175 ± 10
Weight, kg	74 ± 12
BMI, kg/m^2^	24 ± 6
V_DA_, ml	152 ± 19
V_N_, ml	42 ± 6

Values are means ± SD. NS, nonsmokers; S, smokers; XS, exsmokers; BMI, body mass index; V_DA_, anatomical dead space volume based on height (7a), V_N_, nasal volume corresponding to Nasal1 and Nasal2 ROIs derived from individual MRI scans.

In the second part, three male patients who did not require supplemental O_2_ were included, each of whom had received long-time mechanical ventilation through a tracheostomy and then were admitted for weaning. Two of them had chronic obstructive pulmonary disease (COPD; age 59 and 72 yr), and the third patient was recovering from subarachnoid hemorrhage and pneumonia (age 72 yr). This part of the study was approved by the Ethics Committee of Witten-Herdecke University, Germany, and registered under https://clinicaltrials.gov (NCT01509703), and written informed consent was obtained from each subject.

### Nasal high flow.

NHF rates of 15, 30, and 45 l/min without supplemental oxygen were delivered in a randomized order using the AIRVO blower-humidifier and the Optiflow nasal cannula (Fisher & Paykel Healthcare). In the scintigraphy study, NHF was delivered for 30 s (during breath holding). In the tracheotomized nasally breathing patients, NHF was delivered continuously for 10 min. Throughout all studies the mouth remained closed.

### Scintigraphy.

**Fig. 1.**
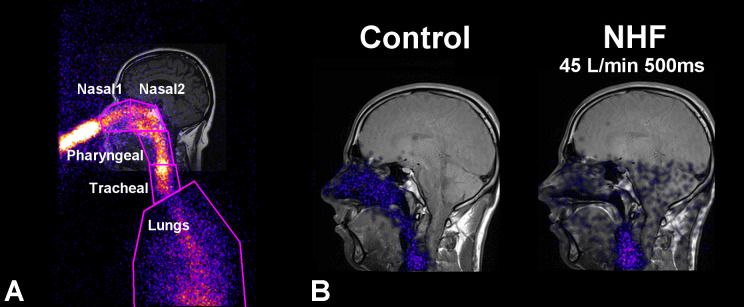
Lateral gamma camera image of nasal ^81m^Kr gas inhalation overlaid on the coronal MRI image of a volunteer during breath holding. *A*: definition of anterior (Nasal1), posterior (Nasal2), pharyngeal, tracheal, and lung ROIs. *B*: visualization of ^81m^Kr gas distribution 500 ms after the application of NHF at a rate of 45 l/min (*right*) compared with the control (*left*) shows fast clearance of the tracer gas in the upper airways. The control measurement without cannula flow shows stable ^81m^Kr gas concentration.

For these experiments the ^81m^Kr gas was generated and a planar gamma camera was used for imaging as described in detail earlier (18). The volunteers filled their upper airways with ^81m^Kr tracer gas through the nasal pillow, and the NHF cannula with the preset flow was inserted into the nose while the volunteer was holding their breath. ^81m^Kr gas activity-time profiles were assessed in five regions of interest (ROI): anterior nasal (Nasal1), posterior nasal (Nasal2), pharynx (space from the soft palate to the larynx), trachea, and the upper lung (Fig. 1*A*). ^81m^Kr gas clearance time constants and half-times were evaluated after correction with the natural ^81m^Kr gas decay (T_1/2_ = 13 s). Nasal clearance rates were evaluated as the ratio of nasal volume (V_N_) and clearance time constant. Nasal volume, comprising the nasal cavity and the nasopharynx (excluding sinuses), was assessed using individual MRI.

### Clearance of anatomical dead space in tracheotomized patients.

Tracheotomized patients were included to assess rebreathing of expired gas from the upper airways. When the weaning from invasive mechanical ventilation was completed, the tracheostomy tube was replaced with a tracheostomy retainer (2). A custom-made probe was placed through the retainer to measure O_2_, CO_2_, and pressure profiles for synchronization with breathing (ADInstruments). Inspiratory volume was assessed with calibrated respiratory inductance plethysmography (RIP; Viasys Services), as described in detail previously (12, 19).

The effect of NHF on the volume of inspired O_2_ and CO_2_ was analyzed for every breath. Inspired O_2_ was calculated in the first 100 ml of inspired volume. Inspired CO_2_ was calculated in the total inspired volume and in the first 100 ml. Arterial blood oxygen saturation ($Sp_{O_{2}}$) and transcutaneous CO_2_ (Tosca; Radiometer) were monitored throughout the study.

### Data analysis.

All data are presented as means ± SD. Differences between groups or application modes were assessed by a two-sided *t*-test using a significance level of *P* < 0.05. Pearson’s coefficient correlation (cc) analysis was then applied to assess the correlation among the study variables.

## RESULTS

### ^81m^Kr gas clearance in healthy volunteers.

After filling the upper airways with ^81m^Kr gas the volunteer was holding his/her breath, and the NHF cannula was attached to his/her nose; this caused immediate purging of the ^81m^Kr gas from the upper airways (Fig. 1*B* and Supplemental Video; Supplemental Material for this article is available online at the *Journal of Applied Physiology* Web site). NHF caused rapid activity decay in the nasal cavity and, as shown in Fig. 1*B*, the nasal cavity was cleared at 0.5 s after applying NHF at a rate of 45 l/min.

**Fig. 2.**
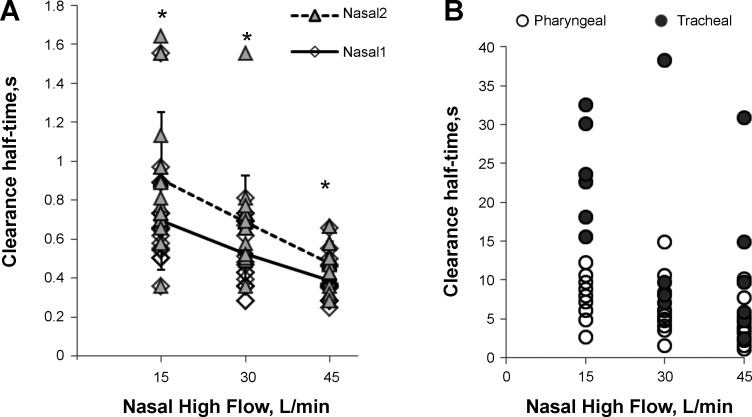
^81m^Kr gas clearance half-times of the anterior (Nasal1) and posterior (Nasal2) nasal cavity (*A*) and in the pharyngeal and tracheal space (*B*) during NHF rates of 15, 30, and 45 l/min. This figure demonstrates flow-dependent clearance (Nasal1 vs. NHF, cc = −0.55, *P* < 0.01; Nasal2 vs. NHF, cc = −0.57, *P* < 0.01) that was always faster in the Nasal1 ROI than in the Nasal2 ROI, which shows a direction of clearance. Data are means ± SD; *P < 0.05, paired *t*-test.

The half-times of ^81m^Kr gas clearance in nasal regions are shown in Table 2 and Fig. 2*A*. For both the anterior (Nasal1) and the posterior (Nasal2) ROIs, there was a decrease in ^81m^Kr gas clearance half-time with an increase of NHF from 15 to 45 l/min (cc = −0.55, *P* < 0.01) in all subjects. Nasal1 ROI cleared faster compared with Nasal2 (*P* < 0.01), and clearance half-times in both ROIs highly correlate (cc = 0.55, *P* < 0.01). There is no correlation between clearance half-times and individual nasal volumes V_N_ derived from MRI scans. Using the time constants for both ROIs and V_N_, the clearance rate in the nasal cavities was calculated: 40.6 ± 12.3, 52.5 ± 17.7, and 72.9 ± 21.3 ml/s during NHF of 15, 30, and 45 l/min, respectively. This demonstrates that there is a significant correlation between clearance rate and NHF (cc = 0.61, *P* < 0.01).

**Table 2.** Half-times T_1/2_ of ^81m^Kr gas clearance in the anterior and posterior parts of the nasal cavity, pharynx, and trachea regions of interest of healthy volunteers during 15, 30, and 45 l/min of nasal high flow

	Half-Time T_1/2_, s		
ROI	NHF 15 l/min	NHF 30 l/min	NHF 45 l/min
Nasal1	0.70 ± 0.26	0.53 ± 0.17	0.39 ± 0.11
Nasal2	0.91 ± 0.34*	0.69 ± 0.24*	0.48 ± 0.11*
Pharynx	7.80 ± 2.96	6.19 ± 3.82	4.43 ± 2.92
Trachea	23.73 ± 6.63	14.30 ± 13.43	10.53 ± 9.85

Values are means ± SD. In all compartments, half-times correlated with NHF (Nasal1, cc = −0.55, *P* < 0.01; Nasal2, cc = −0.57, *P* < 0.01; pharynx, cc = −0.41, *P* < 0.01; trachea, cc = −0.51, *P* < 0.01). Nasal1 and Nasal2, anterior and posterior parts of nasal cavity, respectively.

^*^ *P* < 0.05 Nasal2 vs. Nasal1, paired *t*-test.

In the lower compartments beyond the soft palate, ^81m^Kr gas clearance was also NHF dependent but slower (pharynx, cc = −0.41, *P* < 0.01; trachea, cc = −0.51, *P* < 0.01; Table 2 and Fig. 2*B*), and in some experiments only natural ^81m^Kr gas decay was recorded. Pharyngeal and tracheal clearance half-times correlated with the nasal half times (cc = 0.4, *P* < 0.05). There was no detected ^81m^Kr gas clearance in the lung ROI.

### Rebreathing of expired air during NHF therapy in tracheotomized patients.

**Fig. 3.**
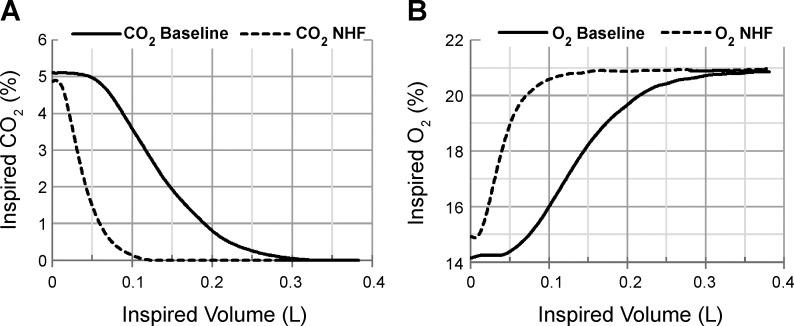
*A*: tracheal CO_2_ concentration plotted against inspired volume of a single breath of a tracheotomized patient demonstrates a decrease of CO_2_ rebreathing during an NHF rate of 45 l/min. *B*: tracheal O_2_ concentration plotted against inspired volume illustrates an increase of O_2_ in the inspired gas during NHF. Both curves of inspired CO_2_ and O_2_ demonstrate maximum differences in the concentration of the gases within the first 0.1 liters (100 ml) of inspired volume.

**Fig. 4.**
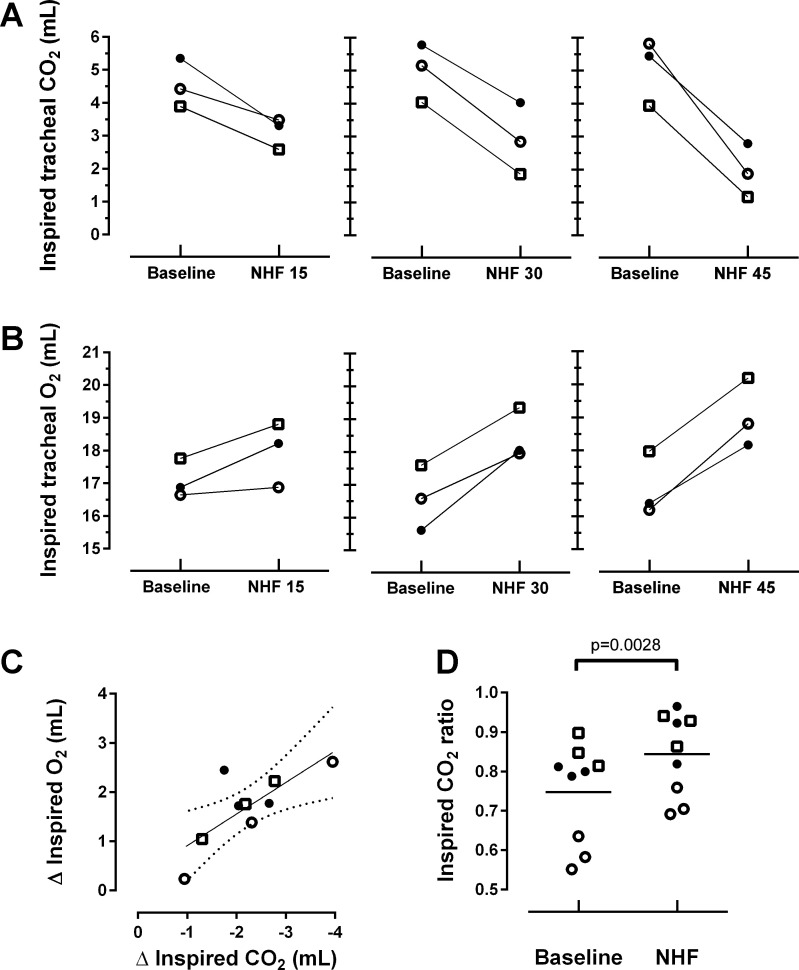
Effect of NHF rates at 15, 30, and 45 l/min on the total inspired tracheal CO_2_ (*A*) and inspired O_2_ (*B*) in the first 100 ml of inspired volume in three patients who are individually represented in the graphs, where the three symbols represent the three NHF rates applied. The data in this figure are presented as means calculated from 2-min intervals. An increase of NHF from 15 to 45 l/min led to a flow-dependent reduction of inspired CO_2_ and a rise in inspired O_2_. *C*: relation between change (Δ) of total inspired O_2_ vs. CO_2_ in the first 100 ml per breath with linear regression (*r*^2^ = 0.59) and 95% confidence intervals. This figure demonstrates that there is a significant correlation between the reduction of CO_2_ and the increase of O_2_ by means of NHF therapy (cc = −0.767, *P* = 0.016). *D*: ratio of inspired CO_2_ in the first 100 ml of tidal volume to the total inspired CO_2_ per breath during baseline ventilation and during NHF (15, 30, and 45 l/min; ratio = 0.84 ± 0.10 vs. 0.75 ± 0.12 for baseline measurements; *P* < 0.01).

An example of a single-breath analysis of inspired CO_2_ and O_2_ at baseline and during an NHF rate of 45 l/min is presented in Fig. 3, *A* and *B*. A summary of the effects of NHF on inspired CO_2_ and O_2_ in the first 100 ml is shown in Fig. 4. In all three patients studied, NHF led to a decrease of inspired CO_2_ and to an increase of inspired O_2_ in a flow-dependent manner (Fig. 4, *A* and *B*). Linear regression analyses between a change (Δ) of total inspired O_2_ vs. CO_2_ in the first 100 ml per breath are presented in Fig. 4*C*. An NHF-induced decrease of inspired CO_2_ correlates with an increase of inspired O_2_ (cc = −0.767; *r*^2^ = 0.59, *P* = 0.016). A ratio between inspired CO_2_ in the first 100 ml of inspired volume and the total inspired CO_2_ grouped by all baselines and NHF treatments is presented in Fig. 4*D*. NHF resulted in a significantly higher ratio during NHF treatment relative to baseline ventilation (0.84 ± 0.10 vs. 0.75 ± 0.12; *P* < 0.01, paired *t*-test). Change of tidal volume, respiratory rate, and minute ventilation as well as $Sp_{O_{2}}$ and tissue CO_2_ throughout the study are presented in Table 3.

**Table 3.** Change of ventilation parameters, peripheral capillary oxygen saturation, and tissue CO_2_ in three patients participating in the study by NHF 15, 30, and 45 l/min during measurement of tracheal gases

	15 l/min		30 l/min		45 l/min	
	Baseline	NHF	Baseline	NHF	Baseline	NHF
*Patient A*						
Tidal volume, ml	332.0	282.6	348.7	300.4	331.5	191.7
Respiratory rate, min^−1^	10.9	12.2	12.3	10.6	12.3	10.8
Minute ventilation, l/min	3.6	3.4	4.3	3.2	4.1	2.1
$Sp_{O_{2}}$, %	96.1	96.4	96.8	96.6	96.9	97.1
Tissue CO_2_, mmHg	32.0	31.8	31.3	31.2	30.7	30.6
*Patient B*						
Tidal volume, ml	366.7	289.7	438.5	364.3	334.6	332.3
Respiratory rate, min^−1^	12.9	14.3	12.2	12.4	15.0	14.8
Minute ventilation, l/min	4.7	4.1	5.4	4.5	5.0	4.9
$Sp_{O_{2}}$, %	92.6	92.2	92.9	92.8	93.5	94.6
Tissue CO_2_, mmHg	48.2	49.1	48.0	48.7	48.7	48.3
*Patient C*						
Tidal volume, ml	290.1	264.1	333.0	255.6	391.1	247.6
Respiratory rate, min^−1^	14.1	13.2	12.2	12.1	14.0	12.3
Minute ventilation, l/min	4.1	3.5	4.1	3.1	5.5	3.0
$Sp_{O_{2}}$, %	96.6	96.5	97.4	97.6	97.0	97.0
Tissue CO_2_, mmHg	39.2	38.5	41.2	40.0	38.3	37.8

All patients had normal respiratory rate and relatively small tidal volume assessed with calibrated respiratory inductance plethysmography. $Sp_{O_{2}}$, peripheral capillary oxygen saturation.

## DISCUSSION

In the first part of the study, dead space clearance by NHF therapy was analyzed in 10 healthy volunteers by the use of ^81m^Kr gas, a radioactive tracer gas, and a gamma camera. The major findings in this investigation are the NHF-dependent reduction of radioactive tracer gas clearance half-times in the upper airways with very fast removal of the tracer gas from the nasal cavities (half-times <0.5 s at an NHF rate of 45 l/min) that confirmed the authors’ model study (18). Furthermore, in various volunteers, significant ^81m^Kr gas clearance was detected in deeper compartments below the soft palate, which could be investigated only in vivo. Rates of NHF in the range of 15–45 l/min were used, which were also used previously (18) and which is common in clinical settings for adults. NHF rates up to 60 l/min were used in patients with acute respiratory failure (28) but cannot be well tolerated by some naïve healthy participants that were found during the preparation of the experiments. In the second part of the study, tracheal O_2_ and CO_2_ breathing profiles in three tracheotomized patients revealed an NHF-dependent increase of inspired O_2_ and a decrease of inspired CO_2_, which confirmed a reduction of rebreathing and supported a hypothesis that NHF reduces dead space.

The ^81m^Kr gas imaging has demonstrated very fast clearance of the tracer gas after the application of high flow through the nasal cannula. The clearance half-times were shorter in the anterior than in the posterior ROIs, demonstrating the direction of clearance, and they were inversely correlated with NHF. Most of the clearance took place in the nasal ROIs with half-times under 1.0 s (Figs. 1*B* and 2*A*).

The clearance study was conducted during breath holding. The effects of respiration on clearance were excluded in this research to avoid the effect of breathing and because of the technical restrictions. In several experiments there was no ^81m^Kr gas clearance below the soft palate (see also Fig. 2*B*). This could be induced voluntarily, since it has been shown that subjects can close their soft palate unintentionally during breath holding, but the mechanism of this reflex is not fully understood (10).

Clearance of ^81m^Kr gas in the lower parts of conducting airways may be of lesser relevance because of very long half-times, as revealed; however, the fact that NHF can produce some clearance even in those deep compartments may suggest a potential increase of the NHF clearance efficiency with a presence of long end-expiratory pauses or opening of the mouth. In other words, clearance of the upper airways by NHF may not be limited by the volume of the nasal cavities.

The results of clearance from nasal cavities are very similar to experiments conducted in upper airway models (18). Faster clearance in the model study can be explained by the lack of restrictions in the reconstructed upper airways compared with those of real human anatomy. Similar to the model experiments used during the current study, the clearance rate was assessed in the same two adjoining nasal ROIs and also showed a linear relationship with NHF. It is nearly doubled (from 40.6 ± 12.3 to 72.9 ± 21.3 ml/s) with an increase of NHF rate from 15 to 45 l/min.

Clearance of tracer gas in the upper airways was further confirmed in tracheal CO_2_ and O_2_ breathing profiles of three tracheotomized patients. The tracheal inhalation profiles plotted for one patient (see Fig. 3, *A* and *B*) show that an NHF rate of 45 l/min reduces the inspired CO_2_ and increases the inspired O_2_ compared with baseline. Profiles of inspired tracheal CO_2_ and O_2_ demonstrate that the maximum difference between the gases is positioned between the first 50 and 100 ml of the inspired volume. NHF resulted in a flow-related reduction of CO_2_ rebreathing (Fig. 4*A*) and an increase of O_2_ in the inspired gas (Fig. 4*B*) with a negative correlation (cc = −0.767; *n* = 9, *P* < 0.05), as further analyzed in Fig. 4, *C* and *D*.

At the end of expiration, conducting airways are filled with gas that typically contains ~5% CO_2_ and 16% O_2_, and at the beginning of inspiration the expired gas is reinspired back into the lungs. NHF delivers fresh air into the upper airways through a pair of nonsealed cannulas, purging the expired gas outside the nasal cavity. There is very little CO_2_ in ambient air (0.04%), and consequently, CO_2_ can be compared in a total inspired volume between the baseline and NHF. Inspired O_2_ is greatly dependent on inspired tidal volume, and in order to accurately measure a relatively small change of O_2_, only a rebreathing portion has to be measured in the inspired volume. The authors chose the first 100 ml to measure a change of inspired CO_2_ during NHF application. A smaller difference between the recorded decrease of inspired tracheal CO_2_ and the increase of inspired tracheal O_2_ can be explained by a calculation of inspired O_2_ in the first 100 ml of inspired gas and the fact that gas was sampled from the trachea into the gas analyzer, prolonging the response time. Inspired CO_2_ is presented in Figs. 3*A* and 4*A* as a total rather than as the first 100 ml per breath, as with O_2_, because of high clinical relevance.

The ratio of CO_2_ in the first 100 ml of inspired air to the total inspired CO_2_, as shown in Fig. 4*D*, resulted in a significantly higher ratio during NHF relative to the baseline (ratio = 0.84 ± 0.10 during NHF vs. 0.75 ± 0.12 at baseline; *P* < 0.01, paired *t*-test). This can be explained by the clearance of expired gas in the upper airways that causes a reduction of the last portion of reinspired CO_2_ measured in the trachea, thereby enhancing the ratio. Therefore, when applying NHF, reinspired CO_2_ primarily results from the first 100 ml of the inspired air, making the difference between the volumes of inspired CO_2_ smaller and shifting the ratio closer to 1.00. It can also be illustrated in Fig. 3*A*, which shows that most of the CO_2_ during NHF is measured within the first 100 ml, consequently increasing the ratio of CO_2_ measured in 100 ml to CO_2_ measured in the total inspired gas volume. The method of the ratio calculation can be recommended for future studies as it is informative and may be used without calibration of inspired volume.

Data on ventilation during the study (Table 3) show a rather small amount of tidal volume measured with RIP in all three patients. RIP was calibrated with a pneumotachograph before and after the experiment and showed very small drift between calibrations, confirming the robustness of the data. Nevertheless, tidal volumes smaller than 250–300 ml with normal respiratory rate may suggest some inaccuracy of the method, which could affect volumes of calculated inspired O_2_ and CO_2_ and lead to an underestimation of the parameters. It is interesting to note that in two experiments, minute ventilation was markedly reduced during NHF while the respiratory rate was within normal values (range 10.6–15.0 min^−1^) and there was no change in blood gases. Reduction of minute ventilation through a decrease of tidal volume may indicate a reduction in the work of breathing without a change in blood gases, which could remain clinically undetected because tidal volume is not measured routinely during NHF therapy. Variability in the ventilation parameters shows that the effect of NHF on ventilation in patients has to be investigated in homogenous groups. The presence of a probe in the trachea may also affect the breathing pattern and is preferably to be excluded in such studies.

### Physiological and clinical implications.

A decrease of rebreathing of CO_2_ by ~1 to 3 ml per breath calculated from the inspired volume with an end-tidal concentration of 5% and a similar increase of inspired O_2_ correspond to a reduction of dead space by 20–60 ml following a rise of the NHF rate from 15 to 45 l/min. This indicates an agreement of data between the scintigraphy part of the study in volunteers and the measurements of inspired gases in the tracheotomized patients. The scintigraphy during breath holding showed the tracer gas clearance at different levels of conducting airways in relation to NHF rates and time. Measurement of CO_2_ and O_2_ in the trachea during respiration confirmed the NHF-dependent decrease of rebreathing of expired air, which is eventually a reduction of dead space.

The reduction of dead space by NHF may increase alveolar volume if tidal volume remains the same. It may also slow down the respiratory rate or reduce tidal volume and minute ventilation, as has been observed in this study and also as previously reported in healthy subjects during sleep (19). Reduction of the respiratory rate is the most frequently described respiratory parameter associated with NHF therapy in adults and children (1, 16, 26), and it is also reported to be a simple and informative predictor of potentially serious clinical events (3). It might be speculated that the reduction of respiratory rate by NHF can be more substantial in patients with an increased respiratory rate. In this study the authors observed very small reduction of the respiratory rate, which was within normal limits, but the small sample size and the study design did not allow for any definitive conclusion. Reduction of dead space may also affect gas exchange: a reduction of arterial CO_2_ (1, 20) and an increase of oxygenation (7, 20) by NHF were shown, although these effects were not evident in this study probably because the NHF application times (10 min) were too short.

The ratio of dead space to tidal volume increases during shallow breathing or when the total physiological dead space is raised because of an increase of alveolar dead space in conditions like emphysema, pulmonary embolism, or acute respiratory distress syndrome (9, 13); this requires an increase of breathing frequency to maintain the same level of alveolar ventilation. For the above-mentioned conditions a small reduction of dead space would lead to a significant improvement in gas exchange resulting in the reduction of minute ventilation or/and the normalizing of blood gas parameters.

Physiological effects and clinical outcomes related to the reduction of dead space during NHF may also be affected by the generated positive airway pressure that can modify breathing patterns and change the efficiency of the dead space clearance. On the basis of the data from the scintigraphy it is also likely that the efficiency of dead space clearance can potentially be increased with the reduction of respiratory rate.

Patients with obstructive and restrictive respiratory disease, as well as stable patients and those in respiratory distress or undergoing respiratory failure, are expected to respond differently to the reduction of dead space by NHF. Nevertheless, an improvement of gas exchange resulting in a reduction of minute ventilation and/or the normalizing of blood gases can be anticipated during NHF therapy.

### Strengths and limitations.

There are two key strengths in the current study. The first is the evaluation of dead space clearance without a breathing component, which is also a limitation and is outlined below. The level of clearance is most efficient in the nasal cavities but may extend below the soft palate; however, this has to be interpreted with caution. The data add weight to the argument that the respiratory support effects of NHF treatment are dependent not only on the NHF rate but also on time; the longer the time during which NHF produces clearance at the end of expiration, the more significant clearance can be expected. The second key strength of the study is that the reduction of rebreathing by NHF was shown via a change of actual gas composition in the inspired air. A correlation between the change of inspired volumes of CO_2_ and O_2_ confirms the validity of the measurements. Elimination of CO_2_ is of primary interest, as a fraction of removed CO_2_ from the expired gas is relatively higher than the added fraction of O_2_, and it is clinically relevant in hypercapnic patients. A role of additional O_2_ as a result of dead space clearance in normoxemic and hypoxemic patients is yet to be determined.

There are limitations to this study, however. The main drawback is that only static clearance rates in the absence of breathing were quantified in the scintigraphy part. There were three reasons to justify the design. First, ^81m^Kr gas has a short lifetime (13 s), and it is a technical restriction to visualize a fast-decaying radioactive tracer gas. Second, tidal breathing would not allow studying the maximum clearance that can be potentially achieved by NHF. Excluded in this study were investigations into the NHF clearance effects during a range of tidal volumes, breathing patterns, as well as openings of the mouth, and positions of the soft palate and vocal cords and the effects of changing the nasal prong size or position; these factors need to be addressed separately in future study designs. Had the authors endeavored to include some of these elements in the current study, they would have had to complicate the protocol significantly and increase the number of patients in the group substantially, who would also have needed to be homogenous to allow adequate quantification of individual responses. The study of three tracheotomized patients was sufficient to demonstrate the NHF-dependent reduction of rebreathing as a physical process—although a large sample size in a controlled trial would be required for the analysis of the above-mentioned parameters, physiological responses, or clinical outcomes of NHF therapy, which need to be studied separately. It is unlikely that an increase in sample size in the study without a change in the design would lead to a valid conclusion on the physiological and clinical effects of NHF therapy as the effects will greatly depend on the baseline parameters and duration of the therapy. Frequent change of NHF rates during a relatively short time is not a desirable study design for assessment of awake, spontaneously breathing patients where an individual voluntary response may affect the results. Also, a maximum NHF rate of 45 l/min was used in this study to repeat the same three flows investigated in a model study (18) and to limit the maximum radioactive daily exposure for the volunteers. In tracheotomized patients there was a risk of noncompletion of the protocol should another NHF rate be added. Apart from the above, the authors could not exclude the fact that some patients would not tolerate higher NHF unless they are in respiratory distress.

In summary, this study has shown effective clearance of the tracer gas by NHF in the upper airways. The clearance is directly related to the NHF rate and time, demonstrating that expired air can be cleared even below the soft palate. The clearance of dead space leads to a reduction in rebreathing of expired air. It may reduce the volume of dead space and increase the alveolar volume, which can result in improvement of alveolar ventilation and gas exchange during NHF therapy.

## Supplemental Data

Supplemental Video Supplemental Video - Supplemental_Video.avi (1.3 MB)

## GRANTS

The study was supported by research grants from Fisher & Paykel Healthcare, Auckland, New Zealand.

## DISCLOSURES

W. Möller received research grants from Pari GmbH, Germany, for studying nasal aerosolized drug delivery, and from Fisher & Paykel Healthcare, New Zealand, for studying the role of nasal high flow in dead space clearance. G. Nilius received research grants from Fisher & Paykel Healthcare, ResMed, Respironics Inc., Philips, Weimann, and Heinen & Löwenstein. S. Feng and S. Tatkov are employees of Fisher & Paykel Healthcare, New Zealand. All other authors declare no conflicts of interest.

## AUTHOR CONTRIBUTIONS

W.M., S.F., U.D., P.B., O.E., O.S., S.T., and G.N. conceived and designed research; W.M., G.C., S.F., U.D., K.-J.F., G.M., and S.T. performed experiments; W.M., G.C., S.F., U.D., O.S., and S.T. analyzed data; W.M., S.F., U.D., S.T., and G.N. interpreted results of experiments; W.M., S.F., S.T., and G.N. drafted manuscript; W.M., S.F., O.S., S.T., and G.N. edited and revised manuscript; W.M., G.C., S.F., U.D., K.-J.F., P.B., G.M., O.E., O.S., S.T., and G.N. approved final version of manuscript.
